# Right Heart Functional Changes in the Acute, Hypercapnic Exacerbations of COPD

**DOI:** 10.1155/2014/596051

**Published:** 2014-06-23

**Authors:** Claudio Terzano, Sofia Romani, Carlo Gaudio, Francesco Pelliccia, Mattia Serao, Antonio Vitarelli

**Affiliations:** ^1^Respiratory Diseases Unit, ALS Respiratory and Critical Care Unit, School of Specialization in Respiratory Diseases, Sapienza University, Viale del Policlinico No. 155, 00161 Rome, Italy; ^2^Department of Heart and Great Vessels Attilio Reale, Sapienza University, Viale del Policlinico No. 155, 00161 Rome, Italy; ^3^Sapienza University, Viale del Policlinico No. 155, 00161 Rome, Italy

## Abstract

*Objective*. To investigate the correlation between respiratory failure and the pulmonary circulation. We focused on anatomical and functional changes of the right heart. *Design*. Clinical investigation. *Methods*. We evaluated 75 patients hospitalized in our respiratory ward for COPD exacerbation. All patients underwent transthoracic echocardiography and measurements of right heart chambers. Moreover all patients underwent blood tests, measurement of blood pressure, evaluation of body mass index (BMI), ECGs, pulmonary function tests, and the Saint George's Respiratory Questionnaire (SGRQ). *Results*. Among 75 patients consecutively hospitalized, 56 patients with a COPD exacerbation were included in our study. We have emphasized the higher value of PAPs and the increased size of right atrial area in severe COPD patients. Significant correlation was observed between low values of PaO_2_ and larger area of the right atrium. The measurement of TAPSE showed a right ventricular dysfunction in all patients but especially in severe COPD patients. We have obtained a significant correlation between TAPSE and arterial blood gas. *Conclusions*. In patients with chronic respiratory failure, blood gas parameters should be considered as negative prognostic factors of right heart failure. Respiratory failure shows a relationship with pulmonary hypertension and with the anatomy and function of the right heart sections.

## 1. Introduction

The chronic obstructive pulmonary disease (COPD) is a chronic disease of the respiratory system characterized by a persistent and irreversible airway obstruction associated with chronic inflammation of the airways and the systemic circulation [1].

The disease is worsening and it is characterized by a tendency to exacerbate with several comorbidities [2]. The WHO estimates that it will become the third leading cause of death in 2020 [3].

Diagnosis of COPD should be considered in the presence of cough and dyspnea, with or without sputum associated with a Tiffeneau index (FEV_1_/VC) ≤ 70 after bronchodilator at a spirometry [1].

The analysis of FEV_1_, together with the use of questionnaires as modified Medical Research Council Dyspnea (mMRC) Scale, body mass index, airflow obstruction, dyspnea, and Exercise Capacity Index (BODE) or St. George's Respiratory Questionnaire (SGRQ) and evaluation of the rate of exacerbation per year will allow us to quantify the seriousness of the disease [4, 5].

COPD often coexists with other comorbidities such as lung cancer, respiratory infections, diabetes mellitus, osteoporosis, increased incidence of atrial fibrillation, arterial hypertension, heart failure, and ischemic heart disease [2, 6, 7].

The study and evaluation of comorbidity leads the clinician to assess their interdependence and then to look for new diagnostic paths in order to identify them early [7].

The prevalence of COPD among individuals with heart failure varies from 20% to 32% of cases and the 10% of patients hospitalized for heart failure also suffer from COPD. On the opposite side heart failure is prevalent in over 20% of patients with COPD [8]. The relative risk (RR) of developing heart failure in COPD patients is 4.5 times higher than that of control individuals without the disease, after adjustment for cardiovascular risk factors [9–13].

Pulmonary arterial hypertension (PAH) is a chronic, progressive disease characterized by an increase in pulmonary arterial pressure above 25 mmHg leading to right heart failure [14]. The pressure increase in the right ventricular chamber is caused by an increase in afterload and generates a progressive remodeling of the right heart. Nowadays it is known that the actual classification of pulmonary hypertension identifies COPD as a cause of the small circle hypertension [14–16].

The causes are the hypoxic vasoconstriction of the pulmonary vascular bed, the inflammatory situation of COPD which in the lungs would lead to such a fibrotic damage to increase vascular resistance, the toxic effects of cigarette smoking, and, finally, an imbalance between the concentrations of endothelin 1 (ET-1) and nitric oxide (NO) which promotes vasoconstriction [17].

The correlation between the progressive remodeling of the pulmonary vascular bed and the increase of the pressures in the right sections of the heart has been studied by many authors in recent years [18–20]. The incidence of pulmonary hypertension in COPD is around 20% but more than 50% in severe chronic bronchitis [21]. The increased interest in the right ventricle has led to several studies about its particular morphology and function which is quite different from the left ventricle [22–25]. The right ventricular chamber is not a passive conduit but an essential part of a circuit characterized by low impedance and high capacitance [26].

The right ventricle is highly susceptible to changes in pressure created by the remodeling of the lung parenchyma in COPD. Tricuspid annular plane systolic excursion (TAPSE) in an apical four-chamber view is a useful surrogate for RV ejection fraction, which indicates the degree of right ventricular function. The level of excursion of the TAPSE, evaluated in *M*-Mode, corresponds with RV ejection fraction (5 mm~20% RVEF, 10 mm~30% RVEF, 15 mm~40% RVEF, and 20 mm~50% RVEF) [27, 28].

The survival of the patient does not seem so much likened to the values of pulmonary artery pressure but to the adaptability of the right sections (loading rate, right atrial pressure, and TAPSE) [28–31]. Pulmonary hypertension and right atrium size in COPD is an important element that we must take into account in the diagnostic program of the patient with COPD, although the clinical manifestations of pulmonary hypertension and right heart failure may be hidden behind the COPD symptoms. Echocardiography with Doppler estimation of pulmonary artery pressure and the size of the right chambers are therefore essential to determine the patient's prognosis.

The aim of our study is to investigate the correlation between respiratory failure and alterations in the pulmonary circulation. We specially focused on anatomical and functional changes of the right heart.

## 2. Methods

From November 2013 to February 2014 we analyzed 75 patients consecutively hospitalized in our respiratory ward (Respiratory Diseases Unit, Policlinico Umberto I, Rome, Italy) for COPD exacerbation.

COPD exacerbations were defined according to the Global Initiative for Chronic Obstructive Lung Disease (GOLD) guidelines [1]. According to GOLD criteria, the subjects with airflow limitation and forced expiratory volume in 1 second (FEV_1_)% predicted ≥ 80 were identified as having mild airflow limitation, 50 ≤ FEV_1_% predicted <80 were described as moderate, and FEV_1_% predicted <50 were described as having severe airway obstruction. FEV_1_ was measured at baseline using a spirometer (Cosmed, Quark PFT, Pavona, Rome, Italy). We included in our study patients with moderate and severe airway obstruction. Hypoxemia was defined by a PaO_2_ < 60 mmHg and hypercapnia was defined by a PaCO_2_ > 45 mmHg on arterial blood gas analysis (ABG). Baseline demographic characteristics and clinical parameters, routine blood chemistry, and ABG were assessed at admission. All patients underwent blood tests, measurement of systolic and diastolic blood pressure, evaluation of body mass index (BMI), ECGs, pulmonary function tests, and disease-specific instrument designed to measure impact on overall health, daily life, and perceived well-being in patients with obstructive airways disease (the Saint George's Respiratory Questionnaire, SGRQ).

All patients underwent transthoracic echocardiography with a commercially available cardiovascular ultrasound system (Vivid E9, GE, Horten, Norway) and measurements of right heart chambers were made according to established criteria [32, 33]. Right ventricular systolic pressure (RVSP) and pulmonary artery systolic pressure (PAPs) were determined by continuous wave Doppler echocardiography [14]. Right atrial pressure was estimated according to caval dimensions [34]. RVSP can be estimated from right atrial pressure using the equation RVSP = 4(VTR)^2^ + RAP, where VTR is peak TR velocity (m/s) and RAP is mean right atrial pressure (mmHg). The mean RAP is estimated using inferior vena cava (IVC) size and reactivity as per American Society of Echocardiography (ASE) recommendations [34]. Estimation of pulmonary vascular resistance (PVR) was determined as previously described [35]. The measurement of TAPSE estimates right ventricle (RV) systolic function by measuring the level of systolic excursion of the lateral tricuspid valve annulus toward the apex in the four-chamber view [28]. A TAPSE > 21 mm/s was consistently observed in the normal population [36]. The echocardiographic assessment was performed during diagnostic tests and patients were not subjected to specific treatment for pulmonary hypertension but only to specific therapy for COPD (*β*_2_-agonists, corticosteroids, chemotherapy, oxygen therapy, and, if necessary, noninvasive mechanical ventilation).

Physical examination was performed and comorbidities were identified on the basis of concomitant therapy and investigations carried out at hospital admission.

Among patients consecutively hospitalized in our respiratory ward, were excluded patients with severe cardiomyopathies, chronic ischemic heart disease, resistant hypertension, atrial septal defect, valvular heart disease, thyroid dysfunction, and severe arrhythmia, patients with severe hydroelectrolyte disorders, cancer patients, and patients with severe systemic complications or previous cardiovascular disease.

In addition, we excluded patients with other causes of primary pulmonary hypertension such as interstitial lung disease, sleep-disordered breathing, and alveolar hypoventilation disorders, other pulmonary diseases with mixed restrictive and obstructive pattern, chronic exposure to high altitude, vasculitis, pneumoconiosis, congenital heart disease, and occlusion of pulmonary veins syndrome.

Written informed consent was obtained from all the participants.

### 2.1. Statistical Analysis

Continuous variables are presented as mean ± standard deviation (SD), and differences were evaluated by paired Student's *t*-test or Wilcoxon test, depending on the shape of the distribution curve. Categorical variables are expressed by count and percentage and compared by *χ* ^2^ or Fisher's exact test when appropriated.

The Spearman coefficient was used for measuring linear correlation between variables.

The probability values are 2-sided; a probability value <0.05 was considered to indicate statistical significance.

Statistical analyses were performed by using the software SigmaStat (San Jose, California). Power analysis was performed using STATA v.11 (College Station, TX).

## 3. Results

Among 75 patients consecutively hospitalized in our respiratory ward for COPD exacerbation, 19 (25.3%) were excluded because of several complications. Three of these died for cardiac complications, four patients were excluded for major metabolic disorders, and two patients died for neurological disease not related to hypercapnia. The remaining ten patients were excluded for iatrogenic complications (two patients), myocardial ischemia (one patient), severe renal failure (three patients), and diagnosis of cancer (three patients). Thus 56 patients with a COPD exacerbation were included in our study.

Participants baseline characteristics are summarized in Table 1.

The subjects studied were more males than females. BMI, blood pressure, blood glucose, and the presence of diabetes mellitus, tobacco consumption, and renal failure did not significantly differ between subjects with moderate COPD and severe COPD.

As showed in Table 2, higher levels of PaO_2_ are prevalent in moderate COPD patients (64.2 mmHg ± 4.6 versus 56.5 mmHg ± 2.96; *P* < 0.05). Similarly higher levels of PaCO_2_ are prevalent in severe COPD patients (72.6 mmHg ± 5.3 versus 55.2 mmHg ± 3.5; *P* < 0.05). In addition we have shown a negative trend regarding the EF% (ejection fraction) in patients with severe COPD but not statistically significant.

**Figure 1:**
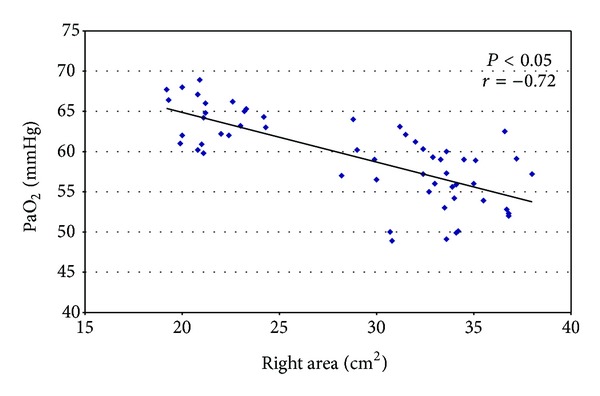
Correlation between low values of PaO_2_ and larger area of the right atrium.

**Figure 2:**
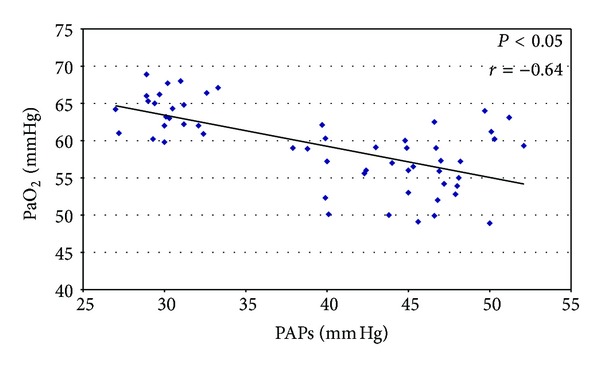
Inverse correlation between PaO_2_ and PAPs.

The assessment of the right heart dimension was available in all 56 subjects. We have emphasized the higher value of PAPs and the increased of right atrial area in severe COPD patients (45.3 mmHg ± 3.5 versus 30.2 mmHg ± 2.3; *P* < 0.05 and 33.3 cm^2^ ± 6.5 versus 21.5 cm^2^ ± 5.2; *P* < 0.05, resp.). An evaluation of the association between right cardiac function and arterial blood gas was performed using multivariable linear correlation. Significant correlation was observed between low values of PaO_2_ and larger area of the right atrium (*P* < 0.05; *r* = −0.72) (Figure 1). The study of PAPs showed values significantly higher in severe COPD patients (45.3 mmHg ± 3.5 versus 30.2 mmHg ± 2.3; *P* < 0.05) and we noted a significant correlation between PaO_2_ in all patients studied (*P* < 0.05; *r* = −0.64) (Figure 2).

**Figure 3:**
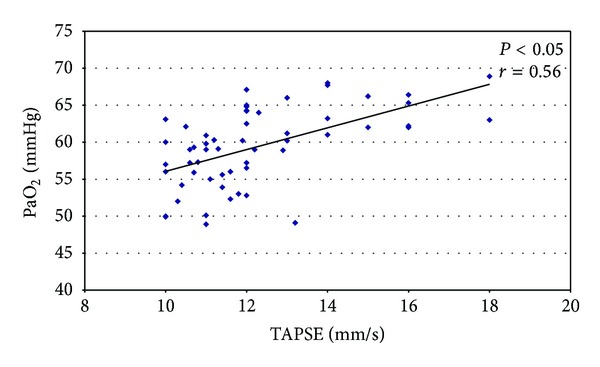
Direct correlation between TAPSE and PaO_2_.

**Figure 4:**
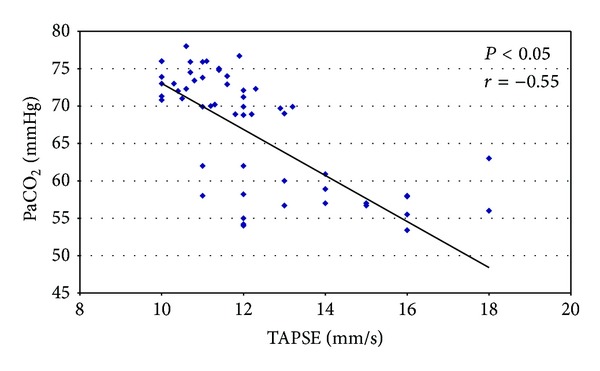
Inverse correlation between TAPSE and PaCO_2_.

The measurement of TAPSE showed a significant right ventricular dysfunction in all patients but especially in severe COPD patients (14 mm/s ± 6 versus 11.2 mm/s ± 2.4; *P* < 0.05) and we correlated the values of TAPSE with arterial blood gas. Thus we have obtained a significant correlation between TAPSE with PaO_2_ (*P* < 0.05; *r* = 0.56) (Figure 3) and TAPSE with PaCO_2_ (*P* < 0.05; *r* = −0.55) (Figure 4).

All patients were subjected to the Saint George's Respiratory Questionnaire but we did not find any significant association with parameters evaluated.

## 4. Discussion

Nowadays the link between chronic respiratory failure in COPD patients and pulmonary hypertension is already widely known. Lung injury induces a progressive reduction in alveolar ventilation. This causes hypoxia which leads to a hypoxic vasoconstriction which increases pulmonary vascular resistance causing hypertension [14, 16, 37].

A study published by Cuttica et al. [38] evaluated 74 patients with COPD. They have examined a possible correlation between the metres walked in the six-minute walking test (6MWT) and FEV_1_, but a significant correlation was not found. This study evaluated a possible correlation between the degree of dyspnea examined by BODE or mMRC and FEV_1_, showing only a link between FEV_1_ and BODE. Similarly in our study we evaluated the degree of dyspnea in patients with moderate or severe COPD with SGRQ but we did not find any correlation statistically significant between the obstruction and the worsening in symptoms at rest. Moreover Cuttica examined the link between PAPs and right atrium area showing a correlation between pulmonary hypertension and anatomical changes of the right heart. In our study we evaluated a correlation between PAPs and arterial blood gas showing how hypoxia in COPD patients causes pulmonary hypertension. Moreover we also stressed a correlation between the atrium area and PaO_2_ showing how the hypoxemia may be considered a negative prognostic factor for right heart failure.

TAPSE is a useful surrogate for right ventricular ejection fraction (EFRV), which indicates the degree of the right ventricular function. The TAPSE estimates the RV systolic function by measuring the level of systolic excursion of the lateral tricuspid valve annulus towards the apex. In a review Bleeker et al. [23] report several studies [27–29] showing an excellent correlation between the TAPSE and RV ejection fraction as assessed by radionuclide angiography. This approach appears to be reproducible and a strong predictor of prognosis in heart failure [23]. In our study we associated TAPSE with PaO_2_ and PaCO_2_ in COPD patients and we have shown that hypoxemia is positively correlated with lower values of TAPSE. At opposite hypercapnia is inversely correlated with TAPSE. This relation was independent of the severity of COPD as measured by FEV_1_ as well as the degree of dyspnea. These correlations further demonstrate the relationship between respiratory failure and right heart failure.

Ghio et al. [27] in a study on the right ventricular systolic function and pulmonary artery pressure sought a better understanding of the coupling between EFRV and PAPs, as it could improve the accuracy of prognostic stratification of patients with heart failure. In fact RV dysfunction is caused by an increase in afterload. Overloading of the pulmonary circle not only causes alterations of the right heart function but also worsens gas exchanges. In fact, we have been able to demonstrate the relationship between ventricular function, expressed indirectly by TAPSE, and hypoxemia.

In conclusion, in patients with chronic respiratory failure, blood gas parameters should not only be considered as parameters to assess the degree of respiratory failure but also as negative prognostic factors of right heart failure. Respiratory failure, in fact, shows a relationship with pulmonary hypertension and with the anatomy and function of the right sections of the heart.

## Figures and Tables

**Table 1:** Patients demographics and clinical characteristics (data expressed as mean ± standard deviation).

Variables	Total (*n* = 56)	Moderate COPD (*n* = 21)	Severe COPD (*n* = 35)	*P* value
Gender: male (*n*, %)	38 (67.8)	16 (76)	22 (62.8)	0.06^b^
Age (years)	79.1 ± 5.1	79.2 ± 5.4	78.2 ± 4.9	0.611^a^
BMI (Kg/m^2^)	26.2 ± 2.6	26.1 ± 3.3	26.5 ± 2.1	0.856^a^
SBP (mmHg)	127.6 ± 16.9	125.8 ± 17.7	135.2 ± 16.2	0.156^a^
DBP (mmHg)	72.4 ± 8.2	70.6 ± 9.8	80.2 ± 6.7	0.097^a^
Fasting blood glucose	119.3 ± 8.7	119.8 ± 7.2	117.3 ± 9.3	0.773^a^
Diabetes mellitus (*n*, %)	41 (73.2)	18 (85.7)	23 (65.7)	0.08^b^
Renal failure (*n*, %)	11 (19.6)	6 (28.5)	5 (14.28)	0.23^b^
Current smokers (*n*, %)	49 (87.5)	15 (71.4)	34 (97)	0.654^a^

^a^Student's *t*-test for unpaired data; ^b^chi-squared test;

BMI: body mass index; SBP: systolic blood pressure; DBP: diastolic blood pressure; COPD: chronic obstructive pulmonary disease.

**Table 2:** Patients main clinical and instrumental parameters (data expressed as mean ± standard deviation).

Variables	Moderate COPD (*n* = 21)	Severe COPD (*n* = 35)	*P* value
FEV_1_ %	66 ± 4.3	44.1 ± 5.2	0.062^a^
pH	7.38 ± 0.02	7.32 ± 0.03	0.2^a^
PaO_2_ mmHg	64.2 ± 4.6	56.5 ± 2.96	0.05^a^
PaCO_2_ mmHg	55.2 ± 3.5	72.6 ± 5.3	0.05^a^
HCO^3−^ mmol/L	30.2 ± 3.2	37.9 ± 5.3	0.256^a^
SO_2_ %	92.5 ± 2.2	89.2 ± 2.7	0.33^a^
EF %	51.2 ± 2.7	36.3 ± 6.3	0.652^a^
TDLVD mm	46.7 ± 4	52.3 ± 3	0.07^a^
PP mm	9 ± 1	8.2 ± 3	0.72^a^
SIV mm	10.4 ± 2	9.6 ± 3	0.725^a^
Left atrial area (cm^2^)	21.7 ± 4	27.3 ± 5	0.062^a^
RVSP mmHg	38.2 ± 2.9	42.4 ± 4.7	0.06^a^
PASP mmHg	30.2 ± 2.3	45.3 ± 3.5	0.05^a^
TAPSE mm/s	14 ± 6	11.2 ± 2.4	0.05^a^
Right atrial area (cm^2^)	21.5 ± 5.2	33.3 ± 6.5	0.05^a^
PVR wood units	1.6 ± 0.4	4.9 ± 1.6	0.07^a^
SGRQ	36 ± 14	41 ± 16	0.06^a^

^a^Student *t*-test for unpaired data;

FEV_1_: forced expiratory volume in one second; PaO_2_: partial pressure of oxygen; PaCO_2_: carbon dioxide partial pressure; HCO^3−^: bicarbonate ion; SO_2_: oxygen saturation; EF: ejection fraction; TDLVD: telediastolic left ventricular diameter; PP: left ventricular posterior wall thickness; SIV: interventricular septum thickness; RVSP: right ventricular systolic pressure; PASP: pulmonary artery systolic pressure; TAPSE: tricuspid annular plane systolic excursion; PVR: pulmonary vascular resistance; COPD: chronic obstructive pulmonary disease; SGRQ: the Saint George's Respiratory Questionnaire.
